# Mitochondrial DNA Sequence Diversity in Mammals: A Correlation between the Effective and Census Population Sizes

**DOI:** 10.1093/gbe/evaa222

**Published:** 2020-10-23

**Authors:** Jennifer James, Adam Eyre-Walker

**Affiliations:** e1 School of Life Sciences, University of Sussex, Brighton, United Kingdom; e2 Department of Ecology and Evolutionary Biology, University of Arizona, Tucson

**Keywords:** diversity, effective population size, census population size, neutral theory

## Abstract

What determines the level of genetic diversity of a species remains one of the enduring problems of population genetics. Because neutral diversity depends upon the product of the effective population size and mutation rate, there is an expectation that diversity should be correlated to measures of census population size. This correlation is often observed for nuclear but not for mitochondrial DNA. Here, we revisit the question of whether mitochondrial DNA sequence diversity is correlated to census population size by compiling the largest data set to date, using 639 mammalian species. In a multiple regression, we find that nucleotide diversity is significantly correlated to both range size and mass-specific metabolic rate, but not a variety of other factors. We also find that a measure of the effective population size, the ratio of nonsynonymous to synonymous diversity, is also significantly negatively correlated to both range size and mass-specific metabolic rate. These results together suggest that species with larger ranges have larger effective population sizes. The slope of the relationship between diversity and range is such that doubling the range increases diversity by 12–20%, providing one of the first quantifications of the relationship between diversity and the census population size.

Significance

What factors influence the level of genetic variation of a species remains one of the most perplexing problems in population genetics. There is an expectation that species with large population sizes should have more genetic diversity but some studies find this relationship and others do not, particularly when the genetic variation in mitochondrial DNA is considered. We have investigated the relationship between genetic diversity in mitochondrial DNA and a measure of the census population size in mammals, using one of the largest data sets considered to date. We find contrary to many previous analyses that DNA sequence diversity is significantly correlated to a measure of census population size. We also quantify this relationship and find that as census population doubles, so DNA sequence diversity only increases by 12%.

## Introduction

One of the central aims of population genetics is to understand why genetic diversity varies between species. However, despite five decades of research and the fact that nucleotide diversities vary by over two orders of magnitude (Lynch and Conery 2003; Leffler et al. 2012), we still have a poor understanding of the factors that affect genetic diversity at the DNA level (Lewontin 1974; Leffler et al. 2012).

Because the level of neutral diversity is expected to depend upon the product of the mutation rate per generation and the effective population size, there has been an expectation that diversity should depend on the census population size. This expectation has generally been met in analyses of nuclear DNA diversity in comparisons between species (supplementary table S1, Supplementary Material online) with two recent exceptions (Romiguier et al. 2014; Mackintosh et al. 2019). However, although there is generally a positive correlation between diversity and measures of population size for nuclear DNA, diversity increases slowly relative to census population size, a pattern that has become known as Lewontin’s paradox, after he pointed out this anomaly (Lewontin 1974); for example, in the early analyses of Soule (1976) and Nei and Graur (1984), it was found that allozyme heterozygosity was linearly related to the logarithm of population size. However, more recent studies have not investigated the relationship between diversity and census population size quantitatively, instead just reporting whether there is a significant relationship between an estimate, or likely correlate of the census population size, and genetic diversity.

In contrast to nuclear DNA, many studies have failed to find a correlation between diversity in mitochondrial DNA and measures of population size between species (supplementary table S2, Supplementary Material online). Even when a correlation exists for the same species for nuclear DNA, a correlation for mtDNA is not necessarily observed (e.g., see Bazin et al. 2006; Singhal et al. 2017). Bazin et al. (2006) ascribed the lack of a correlation between mitochondrial diversity and census population size to genetic hitch-hiking, which might potentially have two effects. First, as Maynard Smith and Haigh (1974) suggested, genetic hitch-hiking might increase in frequency as population size increases if the rate of adaptive evolution is mutation limited. As Gillespie (2000) has shown, this can lead to a disconnect between levels of diversity and population size. Second, hitch-hiking might simply increase the variance in levels of diversity, making it more difficult to observe a correlation, even if one exists. However, an alternative possibility is that there is a negative correlation between the effective population size and the mutation rate per generation which eliminates any correlation between diversity and the effective population size for mtDNA (Piganeau and Eyre-Walker 2009; Allio et al. 2017).

Although many previous analyses have failed to observe a relationship between diversity and measures of population size for mtDNA, they either have tended to look over very broad phylogenetic scales or have limited sample size. Considering organisms over very broad phylogenetic scales might make it difficult to detect any correlation between diversity and population size because many other factors might also vary, including population density and the mutation rate. For example, Allio et al. (2017) have shown that the ratio of mitochondrial and nuclear mutation rates varies substantially between vertebrates and invertebrates, and when this is accounted for, they find a positive correlation between mitochondrial and nuclear diversity across diverse species of animals.

Here, we reconsider the relationship between mtDNA diversity and population size within a phylogenetically limited group of organisms, the mammals, using the most extensive data set compiled to date. Although the vast majority of genetic diversity lies within the nuclear genome, there are several reasons for studying diversity in the mitochondrial genome. First, the population and evolutionary genetics of mitochondria has been of a great interest for many years, principally because it is easy to sequence. Second, understanding the factors that affect the diversity of mitochondrial DNA may increase our understanding of the population genetics of nuclear DNA. Third, studies in which mtDNA is introgressed onto different nuclear backgrounds suggest that a substantial amount of phenotypic variation can be ascribed to genetic variation in the mitochondrial genome (Roubertoux et al. 2003; Dowling, Abiega, et al. 2007; Dowling, Friberg, et al. 2007; Clancy 2008; Montooth et al. 2010; Yee et al. 2013; Latorre-Pellicer et al. 2016). And fourth, parts of the mtDNA have been sequenced in many more species than parts of the nuclear genome.

We investigate whether diversity in mtDNA is correlated to a measure of the census population size, the species range, and we attempt to quantify the relationship between the census and effective population sizes for the first time in a large data set. We also investigate whether it is correlated to a number of life history and demographic variables, as potential correlates of population density and the mutation rate, two other factors that might be expected to affect levels of neutral diversity.

## Results

We collected mitochondrial polymorphism data from 639 mammalian species for which at least four individuals have been sequenced. The average number of individuals sequenced was 15 and the average length of our alignments was 1,300 bp. We also compiled life history and demographic information for many of these species. Variables included in the analysis were range size, absolute latitude, adult body mass, age of sexual maturity, longevity, and mass-specific metabolic rate (MSMR). These were chosen because they either have been shown to be correlated to diversity in previous analyses or might act as proxies for population density or the mutation rate. All of our variables show a significant phylogenetic signal, with Pagel’s *λ* close to one for everything except our two diversity statistics and range (table 1). As a consequence, we used the method of independent contrasts in all analyses (Felsenstein 1985).

**Table 1:** Testing for Phylogenetic Inertia Using Pagel’s *λ*, Using Log-Transformed Data

Trait (Log Values)	Pagel’s *λ*	*P* Value
*π*_S_	0.39	1 × 10^−14^
*π*_N_/*π*_S_	0.40	4 × 10^−12^
Mass	1.0	2 × 10^−306^
Longevity	0.92	3 × 10^−70^
Sexual maturity	0.95	2 × 10^−85^
Mass-specific metabolic rate	0.99	1 × 10^−31^
Range	0.64	4 × 10^−35^
Absolute latitude	0.84	6 × 10^−72^

Note.—The *P* value is from a likelihood ratio test against the hypothesis that there is no phylogenetic signal, that is, *λ* = 0.

### Genetic Diversity

**Fig. 1:**
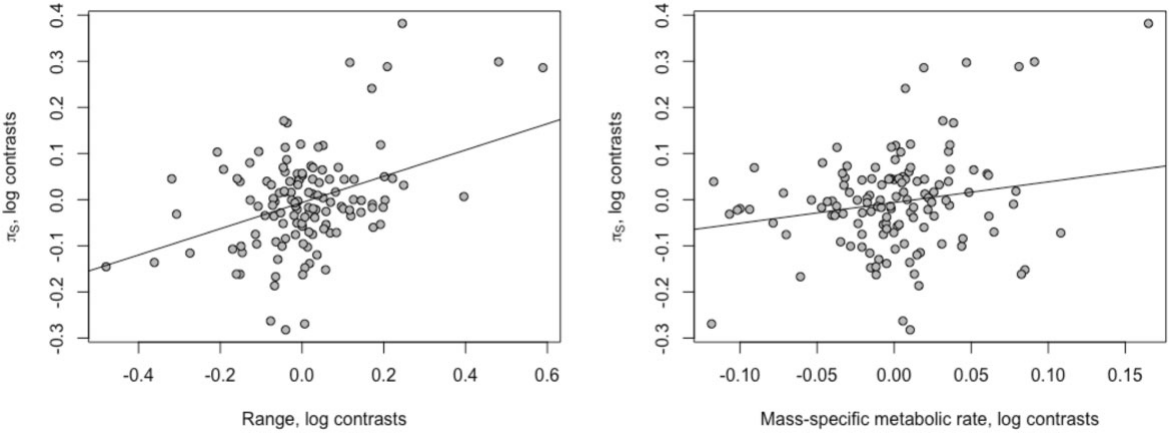
The correlation between *π*_S_ and its two strongest predictors: the global range of a species and the MSMR of a species. The values plotted are phylogenetic contrasts of the log-transformed variables. (The lines shown have the slope from the multiple linear regression model of *π*_S_, including range and mass, range slope = 0.28, *P *=* *1 × 10^−5^, MSMR slope = 0.44, *P *=* *0.024.)

There is little evidence that selection acts upon synonymous sites in mammalian mitochondrial DNA, and hence we use synonymous diversity as a measure of neutral genetic diversity. We find that synonymous nucleotide diversity, *π*_S_, is significantly positively correlated to the geographic range of a species and MSMR (fig. 1 and table 2), and significantly negatively correlated to the absolute latitude, and age at sexual maturity (table 2). However, many of the life history and demographic traits in mammals are correlated; we therefore used a multiple linear regression modeling approach to consider the joint effects of traits on *π*_S_. In the following models, we exclude longevity: this variable is strongly correlated to age at sexual maturity (Pearson’s *R* on log-transformed data = 0.82, *P *=* *2 **×** 10^−47^), and unlike age at sexual maturity it is not correlated to *π*_S_ in a single linear regression. We are also unable to include both mass and MSMR in a single model, because they are very strongly negatively correlated (*R* = −0.88, *P *=* *5 **×** 10^−49^), leading to high variance inflation factors. However, we decided to include MSMR as it is correlated to *π*_S_.

**Table 2:** Results of Correlation Analyses for Two Molecular Evolutionary Traits in Mitochondrial DNA: *π*_S_ and *π*_N_/*π*_S_, with Life History and Demographic Traits

Trait (Log Values)		*n*	Pearson’s Correlation Coefficient	
			*r*	*P*
Mass	*π*_S_	537	−0.039	0.37
	*π*_N_/*π*_S_	466	0.034	0.47
Longevity	*π*_S_	225	−0.056	0.40
	*π*_N_/*π*_S_	202	0.071	0.31
Age of sexual maturity	*π*_S_	238	−0.16	0.011
	*π*_N_/*π*_S_	217	0.029	0.67
Mass-specific metabolic rate	*π*_S_	144	0.20	0.018
	*π*_N_/*π*_S_	129	−0.22	0.012
Range	*π*_S_	556	0.32	2 × 10^−14^
	*π*_N_/*π*_S_	476	−0.21	2 × 10^−6^
Absolute latitude	*π*_S_	556	−0.11	0.013
	*π*_N_/*π*_S_	476	0.060	0.19

Note.— Values are log-transformed before phylogenetic contrasts are calculated. The column *n* gives the number of contrasts available for each correlation.

We find that in a multiple linear regression of all remaining traits, only geographic species range and MSMR are significantly correlated to *π*_S_. Although age at sexual maturity is significantly correlated to *π*_S_ (see table 1), including this variable does not significantly improve the model fit (analysis of variance test on linear models with range and MSMR, and either with or without age at sexual maturity: *P *=* *0.74) and if we remove this variable, our data set increases to 128 species contrasts from 87 species contrasts. Including latitude does not significantly improve the fit of the model (*P *=* *0.75). Overall, a multiple linear regression for *π*_S_ including range and MSMR has an overall adjusted *R*^2^ = 0.20, *P *=* *5 **×** 10^−7^, with both variables being positively correlated to *π*_S_.

Range size and MSMR explain relatively little of the variance in synonymous diversity—respectively 10% and 4.0% (fig. 1 and table 2). The slopes are also shallow. For range, the slope between the contrast in log synonymous diversity and the contrast in log range is 0.16 in a simple regression and this implies that as range size doubles, so diversity increases by just 12%. For MSMR, the slope is 0.54 suggesting that as MSMR doubles so *π*_S_ increases by 45%.

### The Efficiency of Selection

**Fig. 2:**
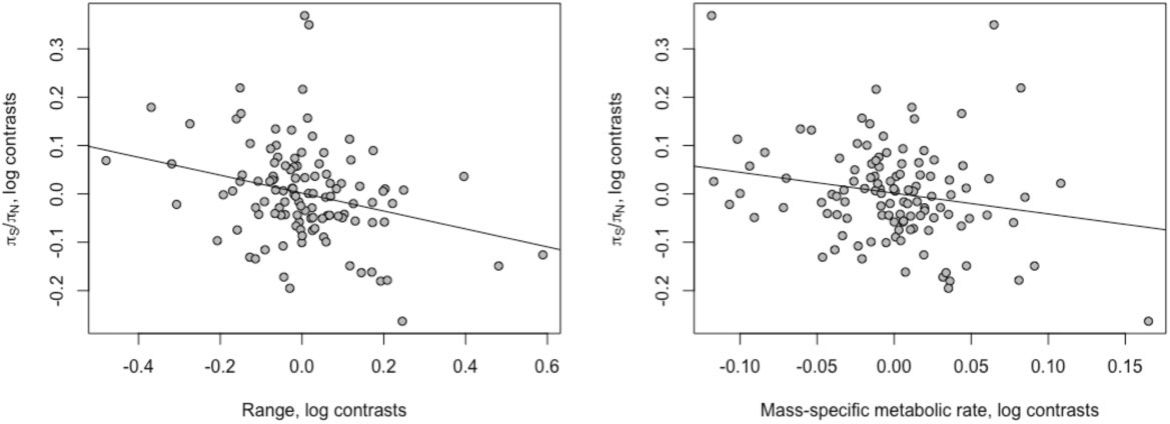
The correlation between *π*_N_/*π*_S_ and its two strongest predictors: the global range area of a species and the MSMR of a species. The values plotted are phylogenetic contrasts of the log-transformed variables. (The lines shown have the slope from the multiple linear regression model of *π*_N_*/π*_S_, including range and mass, range slope = −0.19, *P *=* *0.0033, MSMR slope = −0.43, *P *=* *0.033.)

Neutral genetic diversity is expected to be a product of *N*_e_ and the mutation rate. It seems likely that range is a correlate of census population size and this affects the effective population size. The origins of the correlation between diversity and MSMR are less clear; it might be that species with high MSMR have high mutation rates, but it is also possible that MSMR is related to *N*_e_ in some way, possibly through population density. To investigate the correlation between diversity and MSMR in more depth, and to confirm that the correlation with range is driven by variation in *N*_e_, we investigated whether a measure of *N*_e_, the ratio of nonsynonymous to synonymous nucleotide diversity, was correlated to range, MSMR, and the other variables we have considered. It should be noted that *π*_N_/*π*_S_ is expected to be independent of the mutation rate. We find that *π*_N_/*π*_S_ is only correlated to two variables, range (*r* = −0.21, slope = −0.092, *P *=* *2 × 10^−6^) and MSMR (*r* = −0.22, slope = −0.050, *P *=* *0.012) (table 2 and fig. 2). Note that both correlations are negative, consistent with the patterns seen for *π*_S_ alone; they suggest that *N*_e_ increases with both range size and MSMR, and this leads to an increase in *π*_S_ and a decrease in *π*_N_/*π*_S_. As for *π*_S_, we performed multiple linear regression models for *π*_N_/*π*_S_, including all life history traits; however, range and MSMR remain the only two traits that are significant, with an overall adjusted *R*^2^ of 0.12, *P *=* *0.00024.

### Combining Range and MSMR

The census population depends on both the range of a species and population density. Unfortunately, population density has only been estimated for a few species. However, a potential correlate of density is the reciprocal of the basal metabolic rate of an organism; if a habitat contains a certain amount of energy available to a species per unit area, then dividing the habitat area by the basal metabolic rate should yield an estimate of the maximum possible species density. Hence, a potentially better estimate of the census population size is range divided by basal metabolic rate (MSMR multiplied by body mass). We do indeed find that this measure of population size is more strongly correlated to both *π*_S_ and *π*_N_/*π*_S_ than either is to range (*π*_S_: *r* = 0.43, *P* = 4.0 × 10^−7^; *π*_N_/*π*_S_: *r* = −0.35, *P* = 0.00016) (table 2). The slopes of the relationship between *π*_S_ and *π*_N_/*π*_S_ and the composite measure of population size are 0.28 and −0.20, steeper than we observe for range alone.

### Role of Mutation Rate

Overall, our results suggest that *N*_e_ is an important factor in shaping patterns of mitochondrial molecular evolution. However, mutation rate variation is also likely to affect patterns of mitochondrial diversity. To explore this, we sought to investigate whether a proxy for mutation rate, the rate of neutral divergence, *d*_S_, is correlated to levels of neutral genetic diversity. In this analysis, we used a sister pairs method, which controls for the influence of divergence time on estimates of *d*_S_: our data set was divided into sets of two sister species and an outgroup, which were used to calculate divergence, and we then considered the relationship between the trait contrasts of the sister species in each set, thereby correcting for phylogenetic nonindependence. This data set includes a total of 126 contrasts, however, in order to remove potentially unreliable estimates of *d*s from the data set, we only included contrasts for which 0.00005 < *d*_S_ < 1 for either sister species, resulting in a maximum data set of 98 contrasts. The results do not qualitatively change if, instead of this exclusion step, we improve divergence estimates by including only transversion mutations.

We find that log *d*_S_ is not significantly correlated to any of our life history or demographic traits (similar results were also found by Lanfear and Ho [2010]), nor to any of the polymorphism traits (supplementary table S3, Supplementary Material online). Additionally, if we include *d*_S_ along with range, body mass, and latitude (three factors that we have substantial data for, taking the logs for all variables) in a multiple linear regression model for *π*_S_ (*n *=* *64), we find that including *d*_S_ does not significantly improve our model fit (analysis of variance test, *P *=* *0.86), indicating that *d*_S_ explains little variance in *π*_S_. Excluding *d*_S_, our model results are somewhat similar using this paired method as in our full PIC method as detailed above: range is possibly correlated to *π*_S_ (coefficient = 0.11, *P* = 0.096) though it is not significant. However, we do find a significant effect of latitude on *π*_S_ (coefficient = −0.30, *P* = 0.048). The overall model adjusted *R*^2^ is 0.13, *P *=* *0.011. The discrepancies may occur because in this model we have lower sample sizes and therefore likely lower power.

## Discussion

We have investigated which factors are correlated to levels of synonymous diversity in mitochondrial DNA in mammals. In a multiple regression, we find that diversity is only significantly correlated to MSMR and range size. Both correlations could be driven by a relationship between effective population size and census population size; species with larger ranges have larger census population sizes, and those with higher MSMRs have higher population density. This hypothesis is supported by the correlation between a measure of the effective population size, the efficiency of natural selection, *π*_N_/*π*_S_, and both range size and MSMR.

In a previous analysis of mitochondrial diversity in mammals, Nabholz et al. (2008) found no correlation that survived phylogenetic correction, between diversity and all of the factors considered here, with the exception of latitude. Instead, they found a marginally significant correlation with the substitution rate. However, their data set was considerably smaller than ours—179 species in their largest data set.

### Potential Criticisms

There are a number of criticisms that might be leveled at our study. We have assumed that our sequences are sampled from a single species. However, a positive correlation between diversity and range, and a negative correlation between *π*_N_/*π*_S_ and range might arise if cryptic species groups occupying larger ranges have more species, because both *π*_N_ and *π*_S_ will tend to become dominated by the differences between species. This requires more investigation and is a potential problem with most analyses in this field. We have also assumed that there is no selection upon synonymous codon use. Selection upon codon usage would tend to attenuate the effects of increasing *N*_e_; increasing *N*_e_ would tend to increase diversity but at the same time increase the efficiency of natural selection. Which of these two factors would win out would depend upon the distribution of fitness effects of synonymous mutations. However, there is no evidence of selection on synonymous codon use in mammalian mitochondrial DNA.

### Consistency

It is of interest to ask whether the relationship between *π*_N_/*π*_S_ and range is consistent with the relationship between *π*_S_ and range. Let us assume that synonymous mutations are neutral and nonsynonymous mutations are deleterious with selection coefficients drawn from a gamma distributed distribution of fitness. Under this model $\pi_{S}=2N_{e}u$ and $\pi_{N}=\;2N_{e}ukN_{e}^{-\beta }$, where *N*_e_ is the effective population size of females, *u* is the mutation rate per generation, *k* is a constant, and *β* is the shape parameter of the gamma distribution (Welch et al. 2008). Under this model, we expect the ratio of *π*_N_/*π*_S_ in species 1 relative to species 2 to equal

(1) $$\frac{\pi_{N1}/\pi_{S1}}{\pi_{N2}/\pi_{S2}}={\left(N_{e1}/N_{e2}\right)}^{-\beta }.$$

If we assume that the mutation rate and *N*_e_ are uncorrelated, and *N*_e_ is correlated to some factor *x*, for example, range, then we can estimate the ratio of the effective population sizes from the ratio of the diversities—that is,

(2) $$N_{e1}/N_{e2}=\pi_{S1}/\pi_{S2}=\;{\left(x_{1}/x_{2}\right)}^{\gamma },$$

where *γ* is the slope of the relationship between log(*N*_e_) and log(range). Substituting equation (2) into equation (1) gives

(3) $$\frac{\pi_{N1}/\pi_{S1}}{\pi_{N2}/\pi_{S2}}={\left(x_{1}/x_{2}\right)}^{-\gamma \beta }.$$

An estimate of *γ* can be obtained from the slope of the regression of the contrast in log *π*_S_ against the contrast in log range; for range, this is 0.16. Previously, we have estimated the shape parameter of the distribution of fitness effects (DFE) to be 0.45 in mammalian mitochondrial DNA from the site frequency spectrum (James et al. 2016), and hence we predict the slope between *π*_N_/*π*_S_ and range to be −0.16 × 0.45 = −0.072, similar but slightly lower than the observed slope of −0.092. The excepted slope is less steep than the observed slope, which is consistent with previous observations in both mitochondrial (James et al. 2017) and nuclear (Chen et al. 2017, 2020; Castellano et al. 2018) data sets. Castellano et al. (2018) show that this is to be expected if there is genetic hitch-hiking, because hitch-hiking leads to a nonequilibrium situation in which deleterious nonsynonymous genetic diversity recovers more rapidly than synonymous neutral diversity (Gordo and Dionisio 2005; Do et al. 2015; Brandvain and Wright 2016; Castellano et al. 2018; Chen et al. 2020).

We can also repeat the above analysis for MSMR. The slope of the relationship between the contrast in log *π*_S_ versus the contrast in log MSMR is 0.54, which yields a predicted slope of −0.24, which is much greater than the observed slope = −0.050. The discrepancy suggests that the correlations between MSMR and *π*_S_ and *π*_N_/*π*_S_ might have different origins. Although we do not find a correlation between a measure of the mutation rate, *d*_S_, and MSMR, this may be due to a lack of power; and it remains possible that *π*_S_ increases with MSMR because organisms with higher MSMR have higher mutation rates, but that *π*_N_/*π*_S_ decreases with MSMR due to increased selection on mitochondrial proteins in organisms with high energy demands. In a previous analysis, we found some evidence for this stronger selection; for a given level of synonymous diversity bats, which have very high MSMRs, have lower values of *π*_N_/*π*_S_ than rodents (James et al. 2017).

### Relationship between the Census and Effective Population Size

An important question is whether the slope of the relationship between synonymous diversity and range size reflects the true relationship between effective population size and census population size. There are several potential issues. First, if the effective population size and the mutation rate are correlated then the relationship between diversity and range will either underestimate the slope of the relationship between effective and census population sizes if *N*_e_ and *u* are negatively correlated, or overestimate it if they are positively correlated. Piganeau and Eyre-Walker (2009) have provided some evidence that there is a negative correlation between the effective population size and mutation rate per generation in mammals for mtDNA. Hence, it is likely that the slope of the relationship between the effective and census population sizes has been underestimated; it is steeper than we have estimated, although we cannot say how much steeper it might be.

Second, although range is likely to be a correlate of census size, population density is also very important. However, so long as range and population density are not themselves correlated, then range should give an unbiased estimate of census population. Third, the slope of the relationship between our diversity estimates and census population size may have been underestimated for statistical reasons; error in the independent variable leads to an underestimate of the slope, because very large or small values are partly due to measurement error.

### The Bigger Picture

Several previous analyses of nuclear DNA have also observed that although diversity is correlated to some measure of census population size, diversity increases less rapidly than one would expect if the effective population size was a simple function of census population size; for example, in some of the very first analyses of allozyme diversity, it was found that it increased linearly as a function of log census size (Soule 1976; Nei and Graur 1984). Here, we show that diversity in mitochondrial DNA increases less rapidly than expected—as range doubles so diversity increases by just 12%. There has been considerable debate as to why this is the case. It has been suggested that this scaling might be a consequence of genetic hitch-hiking; as population size increases, so the rate of adaptive evolution increases, increasing the influence of hitch-hiking and hence keeping diversity in check (Maynard Smith and Haigh 1974; Gillespie 2000). For nuclear DNA, this does not seem to be the case. In an analysis of data from 40 animal species, Corbett-Detig et al. (2015) found evidence that hitch-hiking did tend to depress diversity more in species with larger census population size, but the effect was modest; at most they estimated hitch-hiking reduced diversity by 73%, whereas census population sizes vary by many orders of magnitude. In mitochondrial DNA where there is little or no recombination, the effects of hitch-hiking might be more dramatic, and there is some evidence that mitochondrial DNA does undergo substantial levels of adaptive evolution in animals at least (James et al. 2016). If the rate of adaptive evolution scales with population size, for which there is some evidence in nuclear DNA (Gossmann et al. 2012; Rousselle et al. 2020; though see Galtier 2016), then the effective population size is not expected to increase in line with the census population size (Gillespie 2000).

The second possibility is that the mutation rate is negatively correlated to the effective population size, possibly because mutation rates are driven down to a limit set by the power of genetic drift (Lynch 2010; Lynch et al. 2016). There is some limited evidence for this negative correlation within mammals, the group we have considered here (Piganeau and Eyre-Walker 2009), and also across diverse groups of multicellular animals (Allio et al. 2017). However, we find no evidence that a measure of the mutation rate, the synonymous divergence, is correlated to either of our measures of the effective population size. A third possibility is fluctuating census population size; under a model of fluctuating population size, the effective population size is depressed relative to the (arithmetic) average census population size, because it is equal to the harmonic mean of the census population size. However, for this to explain the shallow slope between the effective and census population size, we need to assume that the fluctuations are proportionally bigger for species with large census population size, as suggested by Romiguier et al. (2014), but for which there is little evidence. We are therefore not much closer to understanding why diversity scales as it does with the census population size, although in this analysis we present one of the best estimates of the quantitative relationship between diversity and census population sizes.

The forces that determine the level of genetic diversity in a population remain unclear. For nuclear DNA, many studies find a positive correlation between diversity and some measure of census population (supplementary table S1, Supplementary Material online); the measures of population size in these studies varied widely, from direct measures, such as the number of fish caught, to more indirect measures, such as whether the species is endangered (supplementary table S1, Supplementary Material online). However, two recent studies have failed to find any relationship between diversity and measures of census population size (Romiguier et al. 2014; Allio et al. 2017; Mackintosh et al. 2019). In the first, Romiguier et al. (2014) sequenced the transcriptomes from multiple individuals of 75 multicellular animal species. They found no correlation between synonymous nuclear diversity and two measures of range, the average and maximum distance between GPS records, combining data from their samples and those recorded in the Global Biodiversity Information Facility database (www.gbif.org, last accessed January 07, 2020). However, many of their species are present on multiple land masses, often different continents, so neither of these measures are likely to be correlated to species range. Instead, they found that diversity was strongly correlated to propagule size, the size of offspring when parental care ceases. In contrast, Mackintosh et al. (2019), in an analysis of diversity from 38 European butterfly species, failed to find any correlation between diversity, range, or propagule size; instead, they found diversity was negatively correlated to body size and positively correlated to genetic map length.

In the case of mitochondrial DNA, studies have typically found no correlation between diversity and measures of census population size (supplementary table S2, Supplementary Material online). In part, this seems to be due to variation in rate of mutation obscuring this relationship (Allio et al. 2017), particularly when comparisons are made across phylogenetically diverse groups. Our study, within a fairly restricted group of animals, the mammals, does recover a correlation between diversity and a measure of census population size.

Further work is clearly needed to elucidate the forces that affect genetic diversity, but it may be that there are no universals, and that diversity is determined by different factors in different groups of organisms.

## Materials and Methods

Mitochondrial coding DNA sequences were downloaded from Mampol, a database of mammalian polymorphisms (Egea et al. 2007). Only those species for which there was a minimum of four sequenced individuals were included in this study. Sequences were aligned by eye using Geneious version 7.0.6 (Kearse et al. 2012). Where multiple genes were sequenced for a single species, sequences were concatenated to produce longer alignments. Alignments were then analyzed using our own scripts in order to calculate synonymous nucleotide site diversity, *π*_S_, and a measure of the efficiency of selection, *π*_N_/*π*_S_, a likely correlate of the effective population size. This ratio is undefined if a species has no synonymous diversity, and thus such species were excluded from the analyses of *π*_N_/*π*_S_.

We added life history and demographic information to the species in our data set by using the panTHERIA database (Jones et al. 2009). In this analysis, we focused on six traits: adult body mass (in g), maximum longevity (in months), age at sexual maturity (in days), geographic range (in km^2^), median latitude of geographic range, which was first converted to its absolute value such that in our data set a relationship with latitude represents a relationship with distance from the equator, and MSMR, which was calculated by dividing basal metabolic rate (measured in ml O^2^/h) by the mass (in g) of the individual from which the metabolic rate measurement was taken.

Species cannot be considered as statistically independent datapoints, due to shared ancestry. In order to remove the effects of phylogenetic nonindependence from our data set, we used the method of independent contrasts (Felsenstein 1985). All life history and molecular evolution traits were log transformed, and then phylogenetic contrasts were calculated using the *ape* package in R (Paradis et al. 2004). Our data set using this method has *n* – 1 contrasts, where *n* is the number of species in the data set. The phylogenetic trees used in this study were created using *TimeTree* (Kumar et al. 2017). All analyses were conducted in R. Graphs were created using base R, and the package *jtools*. We also quantified the level of phylogenetic signal in our data set using Pagel’s *λ* (reviewed by Freckleton et al. [2002] and Kamilar and Cooper [2013]), which was calculated with the R package *phylosignal* (Keck et al. 2016).

We also included species divergence data in our results: in order to perform this analysis, we grouped species into triplets, consisting of two sister species, more closely related to each other than any other species in the data set, and an outgroup. By using a sister pair approach, we eliminate the influence of divergence time; that is, the synonymous divergence, *d*_S_ = mutation rate × time of divergence, but the contrast in log *d*_S_ = log *d*_S1_ − log *d*_S2_ = log *d*_S1_*/d*_S2_, which removes the divergence time. To be included in this data set, sister pair species and the outgroup had to have the same mitochondrial gene sequenced, therefore this step reduced the size of the data set considerably. The sequences for each triplet were aligned as before, and then divergence data were calculated using PAML (Yang 2007). In the subsequent analysis, we controlled for phylogenetic effects by conducting our analyses on the relative difference in values between sister species—that is, we calculated species 1_(trait)_/species 2_(trait)_ for every trait for each sister pair, and then considered the relationships between these contrasts. Therefore, the size of our data set using this method is determined by the number of contrasts available, not the number of overall species in the data set. However, as some of the species included in our analysis are relatively divergent, and because mitochondrial mutation rates are very high, our estimates of substitution rates may be unreliable due to the occurrence of sites which are likely to have been hit many times by mutations.

## Supplementary Material

Supplementary data are available at *Genome Biology and Evolution* online.

## Supplementary Material

evaa222_Supplementary_Data
