# Supplementary material for: Mitochondrial DNA Sequence Diversity in Mammals: A Correlation between the Effective and Census Population Sizes
Source: Genome Biol Evol. 2020 Oct 23;12(12):2441–9. doi: 10.1093/gbe/evaa222 (PMC7719226; doi:10.1093/gbe/evaa222)
Supplement: evaa222_Supplementary_Data [file evaa222_supplementary_data.docx]

| Publication | Organism | Molecule | Correlation between diversity and population size | Evidence |
| --- | --- | --- | --- | --- |
| Soule (1976) | Animals | Allozymes | Yes | Significant positive correlation between heterozygosity and crude estimate of log census population size |
| Nevo et al. (1984) | Vertebrates, invertebrates & plants | Allozymes | Yes/no | Generalists have significantly higher heterozygosity than specialists in vertebrates but opposite pattern in invertebrates. No significant difference in plants |
| Nei & Graur (1984) | Prokaryotes & eukaryotes | Allozymes | Yes | Significant positive correlation between heterozygosity and log census population size |
| Hamrick & Godt (1989) | Plants | Allozymes | Yes | Endemic species have significantly lower diversity than narrowly distributed than broadly distributed plants |
| Frankham (1995) | Animals & plants | Allozymes | Yes | Endangered species have lower diversity than non-endangered species in 32 out 38 comparisons |
| Frankham (1997) | Animals & plants | Allozymes | Yes | Island species have lower diversity than related mainland species in 34 of 38 comparisons |
| Spielman et al. (2004) | Mammals | Allozymes & microsats | Yes | Endangered species have lower diversity than non-endangered species |
| Bazin et al. (2006) | Animals | Allozymes | Yes | Various comparisons suggest a relationship between heterozygosity and census population size – e.g. marine fish had higher diversity than freshwater species |
| Nabholz et al. (2008) | Mammals | Allozyme | Yes | Significant correlation between diversity and range size. |
| McCusker & Bentzen (2010) | Fish | Microsats | Yes | Significant positive relationship between diversity and population size, as measured by catch size across 100 species |
| Leffler et al. (2012) | Eukaryotes | Nuclear | Yes | Diversity in arthropods > plants > vertebrates and Drosophila species that are widespread > large-range endemics > small-range endemics |
| Pinsky and Palumbi (2014) | Fish | Microsats | Yes | Overexploited fish have lower diversity |
| Romiguier et al. (2014) | Animals | Nuclear | No | No correlation between diversity and range size, measured as distance between most distantly sampled individuals. |
| James et al. (2016) | Animals | Nuclear | Yes | Mainland species have significantly more nucleotide diversity than related island species. |
| Singhal et al. (2017) | Lizards | RAD | Yes | Species wide diversity correlated to range size and number of museum specimens |
| Grundler et al. (2019) | Lizards | Nuclear | Yes | Positive correlation between diversity and abundance and occupancy across lizard species |
| Mackintosh et al. (2019) | Butterflies | Nuclear | No | No correlations between diversity and range, abundance or range x abundance |

**Table S1.** The relationship between genetic diversity and population size between species for nuclear DNA.

| Publication | Organism | Molecule | Correlation between diversity and population size | Notes |
| --- | --- | --- | --- | --- |
| Avise (1992) | Vertebrates | mtDNA | Yes | Positive correlation between nucleotide diversity and log population size for 18 populations from 12 species |
| Bazin et al. (2006) | Multi-cellular animals | mtDNA | No | Various comparisons suggest no relationship between diversity and census population size; e.g. marine fish do not have higher nucleotide diversity than freshwater fish. |
| Nabholz et al. (2008) | Mammals | mtDNA | No | No correlation between diversity and current range size |
| Nabholz et al. (2009) | Birds | mtDNA | No | No correlation between diversity and current range size |
| Elbe et al. (2009) | Fish | mtDNA | Yes | Three species of endemic surgeon fish are less diverse than related widespread fish |
| McCusker & Bentzen (2010) | Fish | mtDNA | Yes/no | Nucleotide diversity not correlated to catch size, but number of haplotypes and haplotype diversity are correlated across 100 species |
| Delrieu-Trottin et al. (2014) | Fish | mtDNA | No | No difference between endemic and widespread coral fish in diversity across 30 species pairs |
| James et al. (2016) | Animals | mtDNA | Yes | Mainland species have significantly more diversity than island species |
| Singhal et al. (2017) | Lizards | mtDNA | No | Species wide diversity not correlated to range size and number of museum specimens |
| Mackintosh et al. (2019) | Butterflies | mtDNA | No | No correlation between diversity and range, abundance, or range*abundance |

**Table S2.** The relationship between genetic diversity and population size between species for mitochondrial DNA.

| *Trait (Log values)* | *n* | Pearson’s correlation coefficient | |
| --- | --- | --- | --- |
|  |  | r | *p* |
| Mass | 73 | -0.017 | 0.89 |
| Range | 86 | -0.054 | 0.62 |
| Distance from equator | 84 | 0.0011 | 0.99 |
| MSMR | 6 | -0.44 | 0.38 |
| Latitude | 86 | 0.0011 | 0.99 |
| Longevity | 17 | 0.57 | 0.055 |
| Age of sexual maturity | 19 | 0.18 | 0.47 |
| π_s_  π_N_/π_S_ | 98 | 0.0073 | 0.94 |
|  | 84 | 0.0083 | 0.94 |

**Table 3.** The correlation between *d_S_* and other factors.

**Literature cited**

Avise JC. 1992. Molecular population structure and the biogeographic history of a regional fauna: a case history with lessons for conservation biology. Oikos 63:62-76.

Bazin E, Glemin S, Galtier N. 2006. Population size does not influence mitochondrial genetic diversity in animals. Science 312:570-572.

Delrieu-Trottin E, Planes S, Williams JT. 2014. Endemic and widespread coral reef fishes have similar mitochondrial genetic diversity. Proc. Roy. Soc. Ser. B 281.

Eble JA, Toonen RJ, Bowen BW. 2009. Endemism and dispersal: comparative phylogeography of three surgeonfishes across the Hawaiian Archipelago. Marine Biology 156:689-698.

Frankham R. 1997. Do island populations have less hgenetic variation than mainland populations? Heredity 78:311-327.

Frankham R. 1995. Effective population size/adult population size ratios in wildlife: a review. Genet Res 66:95-107.

Grundler MR, Singhal S, Cowan MA, Rabosky DL. 2019. Is genomic diversity a useful proxy for census population size? Evidence from a species-rich community of desert lizards. Mol Ecol 28:1664-1674.

Hamrick JL, Godt MJW. 1989. Allozyme diversity in plant species. In: Brown AHD, Clegg MT, Kahler AL, Weir BS, editors. Plant population genetics, breeding and genetic resources. Sunderland, Massachusetts, USA: Sinauer. p. 43-63.

James JE, Lanfear R, Eyre-Walker A. 2016. Molecular Evolutionary Consequences of Island Colonization. Genome Biol Evol 8:1876-1888.

Leffler EM, Bullaughey K, Matute DR, Meyer WK, Segurel L, Venkat A, Andolfatto P, Przeworski M. 2012. Revisiting an old riddle: what determines genetic diversity levels within species? PLoS biology 10:e1001388.

Mackintosh A, Laetsch DR, Hayward A, Charlesworth B, Waterfall M, Vila R, Lohse K. 2019. The determinants of genetic diversity in butterflies. Nat Commun 10:3466.

McCusker MR, Bentzen P. 2010. Positive relationships between genetic diversity and abundance in fishes. Mol Ecol 19:4852-4862.

Nabholz B, Glemin S, Galtier N. 2009. The erratic mitochondrial clock: variations of mutation rate, not population size, affect mtDNA diversity across birds and mammals. BMC Evol Biol 9:54.

Nabholz B, Mauffrey JF, Bazin E, Galtier N, Glemin S. 2008. Determination of mitochondrial genetic diversity in mammals. Genetics 178:351-361.

Nei M, Graur D. 1984. Extent of protein polymorphism and the neutral mutation theory. Evol. Biol. 17:73-118.

Nevo E, Beiles A, Ben-Shlomo R. 1984. The evolutionary significance of genetic diversity: ecological, demographic and life histroy correlates. In: Mani GS, editor. Evolutionary Dynamics of Genetic Diversity. Berlin: Spinger-Verlag. p. 13-213.

Pinsky ML, Palumbi SR. 2014. Meta-analysis reveals lower genetic diversity in overfished populations. Mol Ecol 23:29-39.

Romiguier J, Gayral P, Ballenghien M, Bernard A, Cahais V, Chenuil A, Chiari Y, Dernat R, Duret L, Faivre N, et al. 2014. Comparative population genomics in animals uncovers the determinants of genetic diversity. Nature 515:261-U243.

Singhal S, Huang HT, Title PO, Donnellan SC, Holmes I, Rabosky DL. 2017. Genetic diversity is largely unpredictable but scales with museum occurrences in a species-rich Glade of Australian lizards. Proceedings of the Royal Society B-Biological Sciences 284.

Soule ME. 1976. Allozyme variation, its determinants in space and

time. In: Ayala F, editor. Molecular Evolution. Sunderland, Massachusetts: Sinauer Associates. p. 60-77.

Spielman D, Brook BW, Frankham R. 2004. Most species are not driven to extinction before genetic factors impact them. Proc. Natl. Acad. Sci. USA 101:15261-15264.
